# Designing Chip-Feed High-Gain Millimeter-Wave Resonant Cavity Antenna (RCA) Array and Optimization of Beam Steering Metasurface

**DOI:** 10.3390/mi16020164

**Published:** 2025-01-30

**Authors:** Abu Sadat Md. Sayem, Karu P. Esselle, Dushmantha N. Thalakotuna, Manik Attygalle, Khushboo Singh

**Affiliations:** 1 School of Engineering, Macquarie University, Sydney, NSW 2113, Australia; 2 Department of Electrical and Electronic Engineering, Rajshahi University of Engineering and Technology, Rajshahi 6204, Bangladesh; 3 School of Electrical & Data Engineering, University of Technology Sydney, Ultimo, NSW 2007, Australia; karu.esselle@uts.edu.au (K.P.E.); dushmantha.thalakotuna@uts.edu.au (D.N.T.); khushboo.singh@uts.edu.au (K.S.); 4 DSTG, Edinburgh, SA 5111, Australia; manik.attygalle@defence.gov.au

**Keywords:** beam steering, high gain antennas, metasurface, near-field, optimization

## Abstract

In this article, a chip-fed millimeter-wave high-gain antenna system with in-antenna power combining capability is presented. A low-profile resonant cavity antenna (RCA) array is fed by multiple spherical dielectric resonators (DRs), demonstrating its multi-feed capabilities. Each of the DRs is fed by two microstrip resonators on a planar circuit board. A printed superstrate is used in the proposed RCA as the partially reflecting superstrate (PRS), which makes the antenna profile small. To increase the directivity and gain, a 2 × 2 RCA array is developed. The demonstrated design shows a prominent peak gain of 25.03 dBi, a radiation efficiency of more than 80% and 3.38 GHz 3 db gain-bandwidth while maintaining a low profile. To steer the beam of the demonstrated 2 × 2 RCA array in a wide angular range with a low side-lobe-level, two planar all-dielectric passive beam steering metasurfaces have been designed and optimized. A detailed analysis of the optimization procedure is presented in this article. This numerical investigation is vitally important for realizing beam steering metasurfaces with suppressed side-lobe-level, wide bandwidth, excellent efficiency and less complexity.

## 1. Introduction

Present-day wireless communication is moving toward millimeter-wave band frequencies due to the requirements of increasing the number of users, high bandwidth and high data rates. However, a millimeter-wave band has the limitation of high path loss, which requires high transmission power to compensate for this loss. Transistor-based amplifiers have limited output power; thus, single amplifiers fail to provide the necessary output power for the system. High output power can be supplied by combining powers from several amplifiers in couplers, but the high coupler loss at the millimeter-wave band compromises the benefit of power combination. Instead of using couplers, phased array antenna topology can be used where each antenna of the array is fed from an amplifier and the total radiated power can be formed to be the combined power from each antenna element. This on-air power combining technique has the drawbacks of large heat production due to the closer location of the amplifiers in the phase array antenna system. Compared to these techniques, the in-antenna power combining method offers a more promising solution for transmitting high power [1,2,3,4]. In this technique, powers from multiple sources are concentrated inside a single antenna footprint, thus eliminating the limitations of coupler losses or the losses from closely spaced amplifiers in phased array topology. This in-antenna power combination technique is utilized in this work for a chip fed antenna system for a millimeter-wave band.

**Figure 1:**
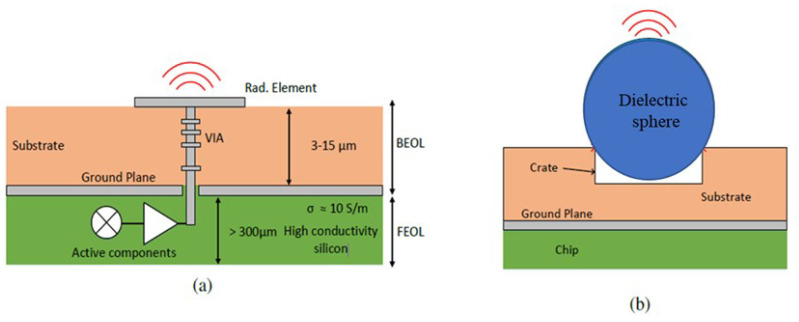
(**a**) Configuration of a standard chip. (**b**) Mounting the spherical dielectric resonator on a crate etched in the chip substrate.

The topology of the chips fabricated with standard processes, such as CMOS, are composed of two segments; the bottom segment of the chip is called Front-End-of-Line (FEOL), which is made with silicon, and the active circuit elements are embedded in this segment. The top segment of the chip is called Back-End-of-Line (BEOL), which contains the passive circuit elements. The substrate (typically SiO_2_) and metallic elements of BEOL are connected to the active components of FEOL through a Vertical Interconnect Access (VIA). Figure 1 illustrates the configuration of a standard chip topology [5]. In this paper, a high gain antenna system is developed that can be directly mounted on the BEOL surface of the chip through a spherical dielectric resonator directly mounted on a shallow crate etched in the substrate of the BEOL (see Figure 1b) and fed from the active components located in the FEOL through the VIA. In the proposed design, electromagnetic energy from several sources are combined inside an RCA array before transmitting through the radiating element of the antenna. The installation of a spherical resonator in the shallow crate ensures high positioning accuracy and stable attachment of the spherical resonator to the chip. This chip-mounted spherical resonator acts as the feeding element of the proposed antenna system. Such an in-antenna power combing technique is highly efficient in millimeter-wave, low-loss, high-power applications.

There is a growing need to dynamically steer the beam of high-gain antennas. Among many applications in the existing and upcoming wireless communications, the most prominent application areas of high-gain beam steering antennas are automotive radar systems, point-to-point and point-to-multipoint communication terminals [6], and on-the-move connectivity [7].

Beam steering of high gain antennas can be accomplished by mechanically moving antenna components [8], liquid crystals [9], digitally controlled transmit arrays [10,11], in-plane translation of lenses with fixed antenna feeds [12], leaky-wave antennas [13], and phased array topology [14]. The phased array antenna topology provides quasi-continuous beam steering for high gain antennas at mm-wave frequencies, but it suffers from the limitations of high loss, poor efficiency and high cost [15,16,17].

Apart from the conventional mechanically movable reflector dishes and electronically steered phased arrays, recently, passive electromechanical beam steering systems have become popular due to their simplicity, wide-range steering and low cost [8,18,19]. A beam steering system utilizing planar phase transformation surfaces mounted in the far-field region (nearly eight wavelengths apart from the antenna aperture) of the fixed-beam antenna is reported in [18]. The topology of this phase transformation surface was motivated by the optical Risley prism [20] and its microwave counterpart (dielectric wedges) [21]. The work in [8] demonstrated that the phase transformation metasurfaces can be placed in the near-field region instead of the far-field region of the fixed-beam antenna, and similar steering performance can be achieved with a lower height of the system. This novel technique introduced a new branch of beam steering development called near-field metasteering, otherwise known as metalenses. This methodology was later followed by [19] to develop two all-metallic phase transformation metasurfaces for high-power applications. Ref. [22] developed a phase gradient metasurface lens by placing a modulated circular disk unit cell on the top of the radiator to control the radiated beam, and this improved the gain with a decrease in beam width. Refs. [23,24] developed a beam tilting system by using a phase-modulated metasurface and negative indexed metasurface loading. A wide beam scanning metasurface with improved gain by controlling the beam of a substrate-integrated slot-array antenna was developed in [25]. Ref. [26] developed a bifocal Fresnel lens by using a polarization-sensitive metasurface.

Phase transformation metasurfaces located in the near-field region can steer the beam within a large conical region. These methods are efficient, suitable for high-power applications and low in cost. However, very limited literature is found on optimizing the metasurfaces for an improved performance. Motivated by the future prospects of beam-steering, high-gain, high-power, chip-fed antennas and the limited research in the optimization of metasurfaces, this article presents an all-dielectric metasurface designed and optimized for millimeter-wave band applications. The all dielectric topology offers the benefit of low loss, design simplicity, ease of fabrication and low cost. Moreover, the phase transformation is achieved by implementing a series of holes with varying radi in the dielectric slabs instead of varying the height of the dielectric. This method significantly reduces the height of the metasurface. The demonstrated optimization technique offers a promising solution for designing metasurfaces with better beam steering performance with reduced side-lobe level, large bandwidth and high efficiency.

## 2. Antenna Topology and Design

### 2.1. Configuration of the Antenna

**Figure 2:**
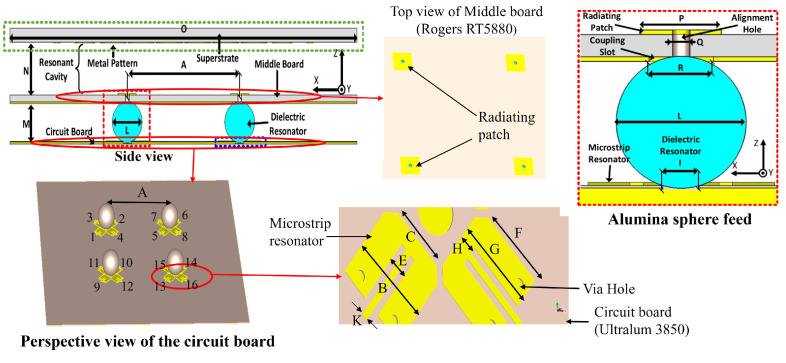
Geometry of the proposed antenna.

Figure 2 shows the configuration of the proposed antenna. The proposed antenna is an RCA with a printed superstrate. A partially reflecting superstrate (PRS) modifies the phase front of the transmitted wave and thus improves the directivity of the antenna. Superstrates are classified as printed and non-printed; non-printed superstrates are constructed by dielectrics where phase correction is implemented by varying the permittivity [27] or the thickness [28] of the dielectrics, while printed superstrates are constructed by printing metallic patterns on the dielectric [29]. In the demonstrated design, a printed superstrate is used where disk-shaped metallic patterns are printed on the dielectric. The RCA is fed by a 2 × 2 square microstrip patch array. These square patches are printed on a middleboard. The substrate of the middleboard is Rogers RT5880. The bottom surface of the middleboard has cladding, which serves as the ground plane for the RCA. Square coupling slots are etched in the cladding exactly underneath each patch to provide electromagnetic coupling between the patch radiator and the dielectric sphere. For proper alignment of the spheres with respect to the middleboard, holes are drilled in the dielectric and through the square patches.

In the demonstrated design, four alumina spheres are used as the feeding elements of the antenna where the spheres are mounted on a thin circuit board. Utilization of spherical DRs are an effective method for feeding such types of millimeter-wave chip-fed antennas because of their perpendicular radiation, excellent radiation efficiency and precise alignment to the circuit board [30,31]. The selected material for the bottom circuit board is Ultralam 3850. Circular crates etched in the circuit board hold the spheres. The alumina spheres have a 3 mm diameter, and each resonates at 34.5 GHz. Four open-ended λ/4 microstrip resonators are used to excite each sphere, where λ is the guided wavelength. Among four microstrip resonators of each sphere, an opposite set of microstrip resonators are fed in opposite phases with the same magnitudes. For impedance matching, each microstrip resonator is shorted to ground through a pair of holes. From the demonstrated antenna topology, it is explicit that there is a total of sixteen microstrip resonators to feed the RCA; thus, power from sixteen sources are combined inside the cavity of the RCA and radiates through the PRS. In actual chip feed applications, the circuit board will be the substrate of the BEOL and each microstrip resonator will be directly fed from a power source located in the FEOL through VIA. Therefore, it can be concluded that the proposed design can be an efficient form of an in-antenna power-combing technique for chip-feed integrations.

**Figure 3:**
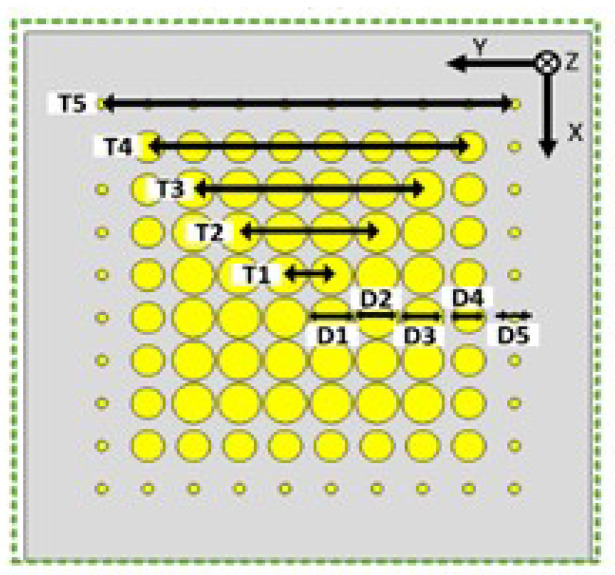
Printed superstrate.

The superstrate of the RCA is shown in Figure 3. The dielectric material for the superstrate is TMM4, whose bottom surface has a printed pattern that consists of circular metallic disks with gradually reduced diameters from the center toward the edge. This configuration imposes more phase delay to the field at the center of the superstrate compared to the edge, incorporating a uniform phase front and enhancing directivity.

Computational analysis of the RCA was accomplished in CST Microwave Studio 2020. The dimensions of the antenna element and superstrate, presented in Figure 2 and Figure 3, are shown in Table 1.

The properties of the different dielectric materials used in different parts of the antenna are mentioned in Table 2.

### 2.2. Performance Characterization

**Figure 4:**
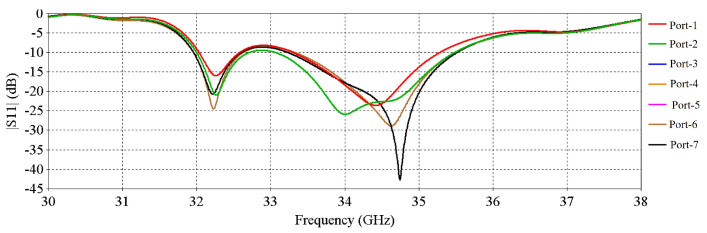
S-parameters of the input ports.

Figure 4 demonstrates the simulated active S-parameters (magnitude of input reflection coefficient) of the input ports. There are a total of 16 input reflection co-efficient results for 16 ports (excited with 50-Ω discrete ports), but for clarity, 7 results are shown in Figure 4 as the results for some ports are exactly similar. The active S-parameters results reveal that this multi-fed RCA maintains impedance matching over a wide bandwidth.

**Figure 5:**
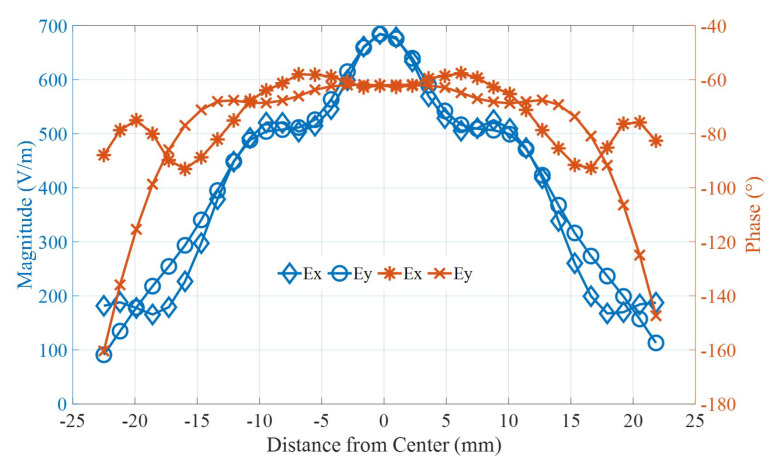
E-Field magnitude and phase response of the printed PRS in X-cut plane.

Figure 5 demonstrates the magnitude and phase response of the printed PRS. Here, only the results for the cut plane in the X direction is shown. Other planes give similar results and are, therefore, excluded to avoid repetition.

**Figure 6:**
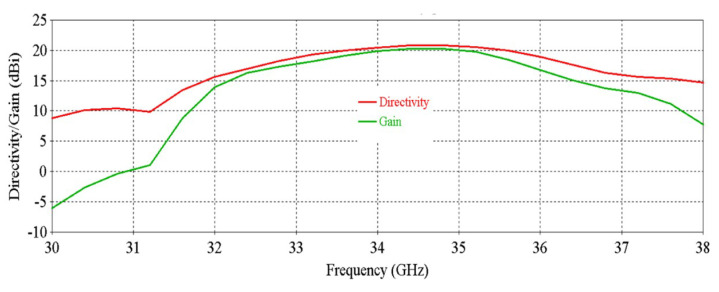
Directivity and gain vs. frequency of the antenna.

**Figure 7:**
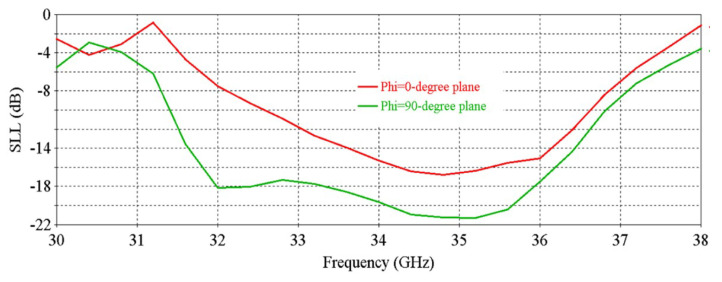
SLL vs. frequency of the antenna.

Figure 6 shows that the antenna achieves a peak directivity of 20.83 dBi at 34.5 GHz. The 3 db directivity bandwidth is 3.77 GHz (32.63–36.4 GHz). From Figure 6, it is observed that the antenna achieves a peak gain of 20.33 dBi at 34.5 GHz with a 3 db gain bandwidth of 3.02 GHz (32.84–35.86 GHz). Figure 7 shows the side-lobe-level (SLL) of the antenna in two orthogonal planes (phi = 0° and phi = 90° planes).

**Figure 8:**
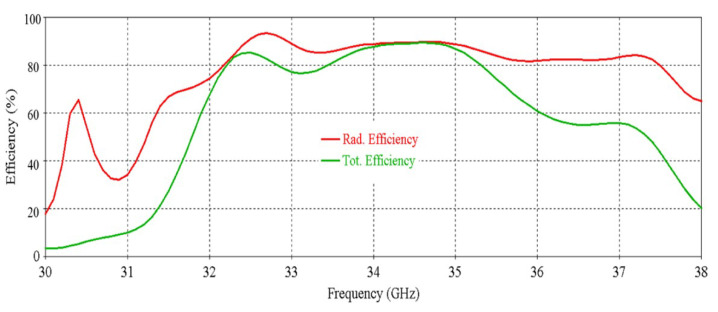
Efficiency vs. frequency of the antenna.

The simulated efficiency of the antenna is shown in Figure 8. It can be seen that the antenna maintains more than 70% radiation efficiency from 32 GHz to 35.5 GHz. At 34 GHz, the efficiency of nearly 90% is observed.

The efficiency of the antenna was calculated from the following equation:

(1) $$\eta_{total}\;=\;\eta_{rad}{\;\times \;(1-|\Gamma |}^{2})$$

where $\eta_{rad}$ is the radiation efficiency, $\eta_{tot}$ is the total efficiency and Γ is the reflection coefficient, which represents the impedance mismatch. The total efficiency is the ratio of the radiated to stimulated power (from all the sources) of the antenna, it takes into account the impedance matching, whereas, the radiation efficiency does not consider the impedance matching.

**Figure 9:**
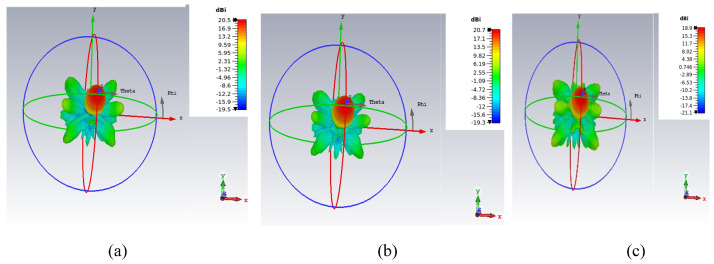
The 3D radiation patterns—(**a**) 32 GHz, (**b**) 34 GHz, (**c**) 36 GHz.

Figure 9 shows the 3D radiation pattern at 32, 34 and 36 GHz. It shows that the maximum radiation is pointing toward the broadside with side-lobe-levels below 8 dB.

From the above numerical analysis, it is revealed that the antenna achieves excellent matching bandwidth, 3 dB directivity bandwidth (3.77 GHz), 3 dB gain bandwidth (3.02 GHz) and promising efficiency in the entire operating band (32 GHz to 35.5 GHz).

## 3. Phased Array Topology

**Figure 10:**
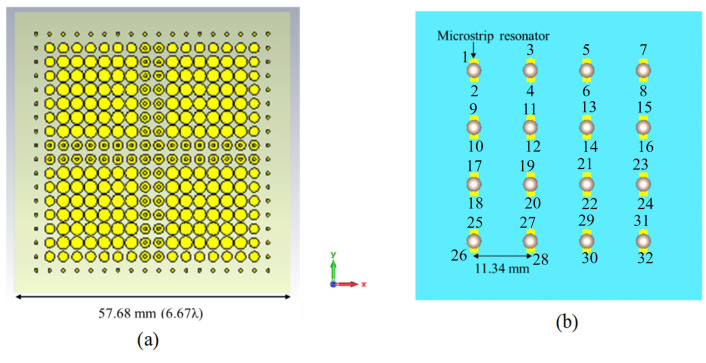
The 2 × 2 RCA array—(**a**) top view, (**b**) feed topology.

To increase the directivity and bandwidth, RCA array technology is implemented. In this work, a 2 × 2 RCA array is used. The top view of the RCA array configuration is illustrated in Figure 10. In the demonstrated RCA array topology, different elements of the array are combined into the same substrate, thus simplifying the assembly and the feeding. In the proposed array technology, two outermost columns and rows of the metallic disks of each element of the superstrate are overlapped (Figure 10a), which substantially reduces the grating lobes. Here, the dimensions of each array elements are the same as the previous single-element antenna, as shown in Figure 3 and Table 1. The separation between the spheres (A) is slightly increased to 11.34 mm to maintain an equal distance between consecutive spheres. The footprint of the antenna is 57.68 mm × 57.68 mm. Figure 10b shows the dielectric spheres of the array. There are in total 16 spheres and each is fed from two vertical microstrip resonators. Therefore, there are in total 32 input/output ports for this 2 × 2 RCA array.

### Performance of the RCA Array

**Figure 11:**
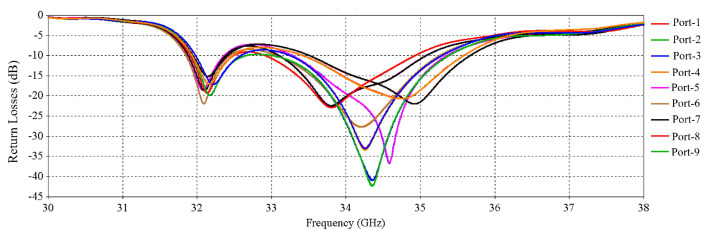
S-parameters of the 2 × 2 RCA array.

The numerical performance of the RCA array was investigated in CST Microwave Studio 2020. Figure 11 shows the simulated active input match at each port. There are in total 32 results for 32 ports, but for clarity, 9 results are shown in Figure 11 as some results are exactly the same, so these are skipped here. The S-parameter results indicate good matching from 31.8 to 35.5 GHz, except for some relatively poor matching for some ports at 33 GHz.

**Figure 12:**
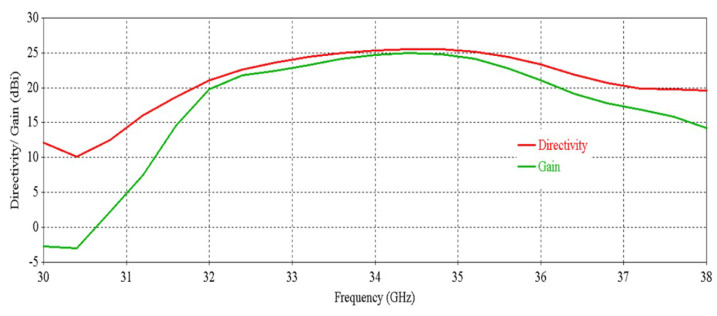
Directivity and gain vs. frequency of the 2 × 2 RCA array.

**Figure 13:**
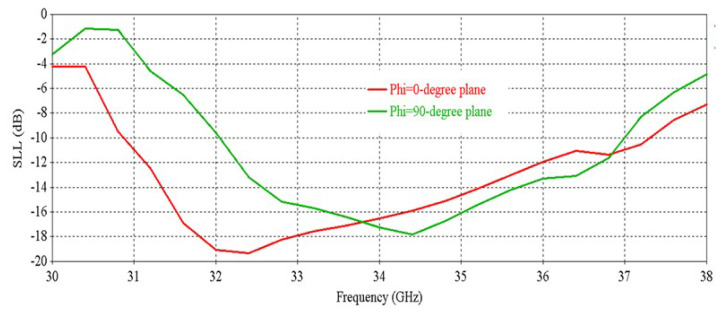
Side-lobe-level (SLL) vs. frequency of the 2 × 2 RCA array.

Figure 12 shows that the antenna achieves a peak directivity of 25.6 dBi at 34.5 GHz, whereas for a single element, the directivity was 20.83 dBi. Therefore, the directivity has been increased by 4.77 dB. The 3 db directivity bandwidth of the 2 × 2 RCA array is 3.85 GHz (32.37–36.22 GHz), whereas for individual elements, the 3 db directivity bandwidth was 3.77 GHz. Therefore, 3 db directivity bandwidth has been increased by 0.08 GHz in the 2 × 2 RCA array. From Figure 12, it is observed that the antenna achieves a peak gain of 25.03 dBi at 34.5 GHz with a 3 db gain bandwidth of 3.38 GHz (32.44–35.82 GHz). For a single element, the peak gain was 20.83 dBi and 3 db gain bandwidth was 3.02 GHz. Therefore, the peak gain is increased by 4.2 dB, and the 3 db gain bandwidth is increased by 0.36 GHz. Figure 13 demonstrates the excellent side-lobe-level (SLL) of the RCA array in two orthogonal planes (phi = 0° and phi = 90° planes).

**Figure 14:**
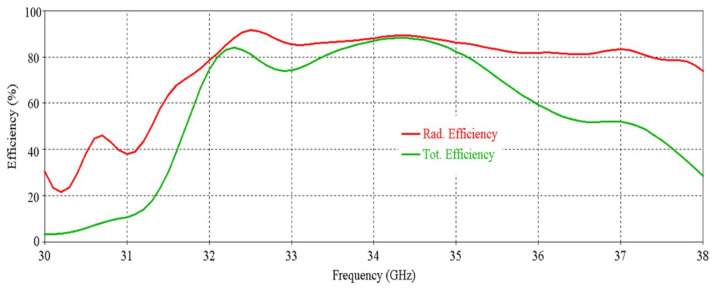
Efficiency vs. frequency of the 2 × 2 RCA array.

The simulated efficiency of the RCA array is shown in Figure 14. It can be seen that the antenna maintains a radiation efficiency of more than 80% from 32.1 GHz to 37.3 GHz. A decrease in total efficiency is seen at 33 GHz and after 35 GHz, and this is due to poor matching at these frequencies.

**Figure 15:**
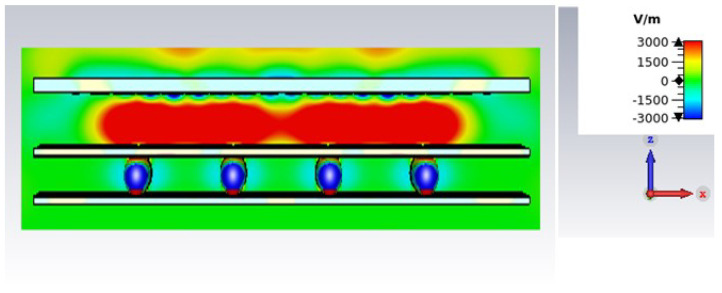
E-field distribution of the 2 × 2 RCA array at 34.5 GHz.

The E-field distribution of the RCA array is shown in Figure 15. There is overlapping hotspots between array elements, which indicates good radiation characteristics of the array.

**Figure 16:**
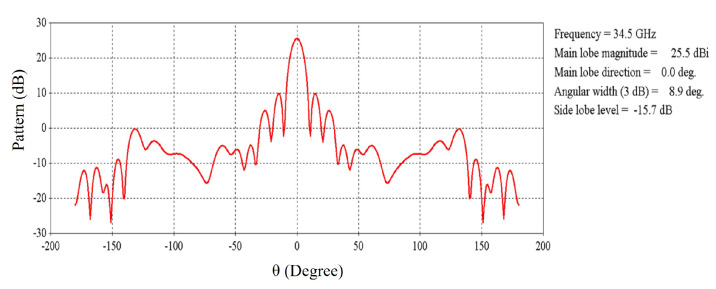
Radiation pattern of the 2 × 2 RCA array at 34.5 GHz.

The far-field radiation pattern of the RCA array at 34.5 GHz is shown in Figure 16. It is exhibited that the maximum radiation pointing toward the broadside is 25.5 dBi with side-lobe-levels lower than 15.7 dB.

## 4. Beam Steering Metasurface Design

To tilt the beam at the desired angle, a planar phase gradient all-dielectric metasurface is designed. In the demonstrated design, a pair of planar near-field phase gradient metasurfaces are utilized to steer the beam. When two metasurfaces are rotated together in the same direction keeping no change to their relative positions, a conical scan of the main beam is achieved. On the other hand, the beam is steered in the elevation plane only when both metasurfaces are rotated at the same angle in the opposite direction to each other. Therefore, beam steering is possible in any direction within the cone by changing the relative position of the metasurfaces [32].

### 4.1. Unit Cell Model

**Figure 17:**
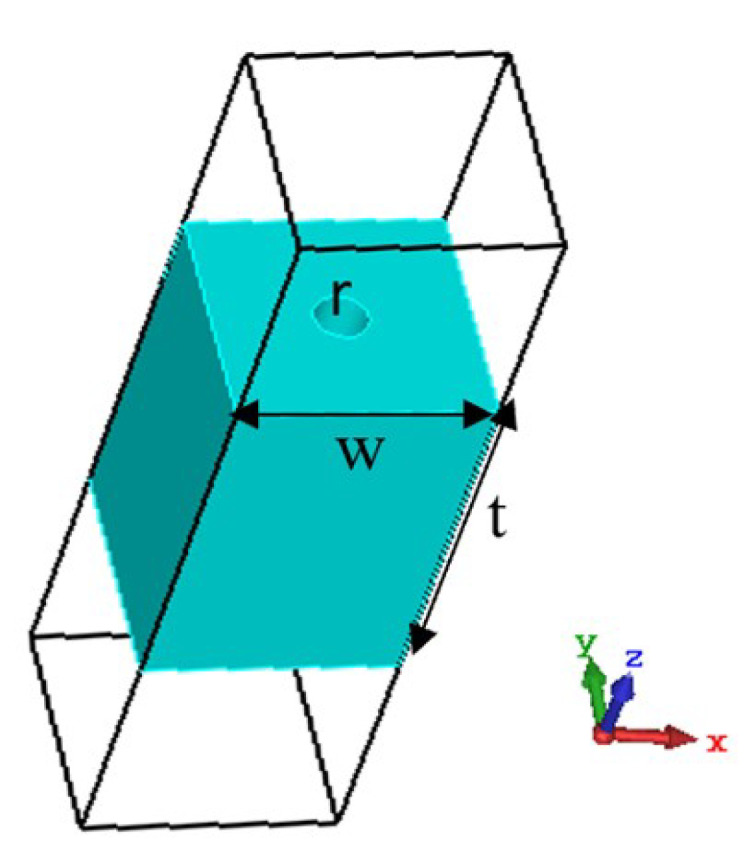
Unit cell of the proposed metasurface (*w* = 2.85 mm, *t* = 4.5 mm).

Figure 17 shows the unit cell of the proposed metasurface. The unit cell is a square dielectric block with a width *w* and thickness *t*. All four different dielectric materials are combined to form the whole metasurface structure with a thickness of 4.5 mm. A circular through hole with radius *r* is made in the middle of the dielectric; by varying the radius of the hole, the effective dielectric constant of the cell can be changed, which in turn changes the phase shift of the electromagnetic wave passing through the dielectric block. With the combination of different dielectric materials and radiuses of the holes, a full 360° phase shift can be achieved through this cell configuration. In this design, four different dielectric materials are used to achieve the 360° phase-shift range, which are:

PREPERM ABS300 ($\epsilon_{r}$ = 3, tanδ = 0.0046)

PREPERM ABS450 ($\epsilon_{r}$ = 4.5, tanδ = 0.0042)

PREPERM ABS650 ($\epsilon_{r}$ = 6.5, tanδ = 0.0035)

PREPERM ABS1000 ($\epsilon_{r}$ = 10, tanδ = 0.003)

**Figure 18:**
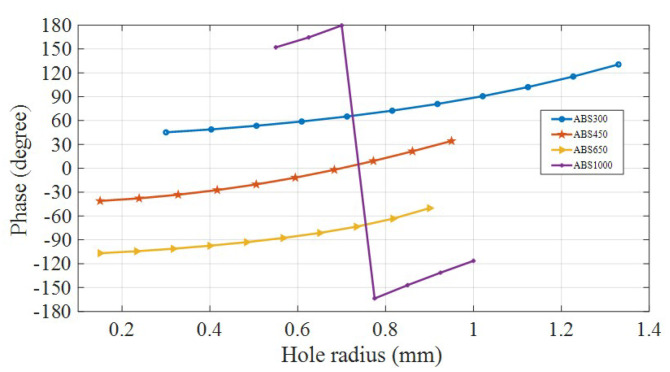
Unit-cell -normalized phases.

These dielectric materials are used to realize the near-field phase gradient metasurface. Normalized phases achieved with these four dielectric materials with varying radiuses of through holes between 0.1 mm to 1.4 mm are plotted in Figure 18.

### 4.2. Metasurface Development from Unit Cell Model

**Figure 19:**
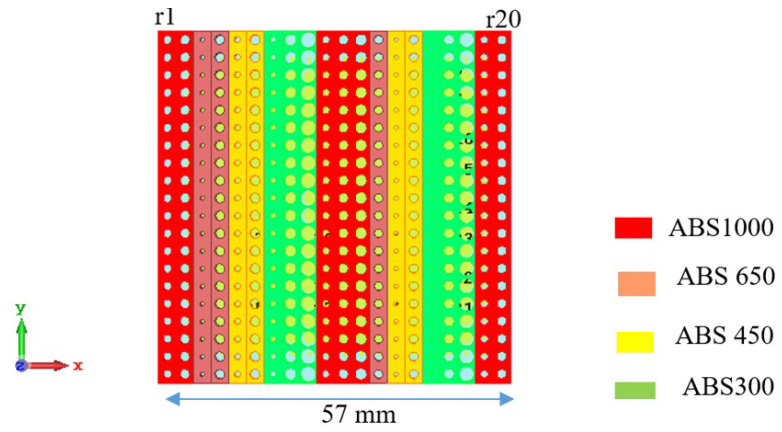
Metasurface configuration.

The proposed metasurface is a near-field phase gradient metasurface, which is designed for the tilt angle of 20°. From the theory of the phase gradient metasurface, it is known that to obtain a beam tilt of $\theta^{{}^{\circ}}$ at a wavelength of $\lambda_{0}$ in a 1-D array, the progressive phase delay Δϕ required between adjacent elements is given by the following relationship [8,32]

(2) $$\Delta \phi \;=\;\frac{2\pi }{\lambda_{0}}d\sin\theta$$

where *d* is the center-to-center distance between adjacent cells. Therefore, to tilt a normal beam by 20°, a metasurface requires a progressive phase shift (Δϕ) of 41° between adjacent cells for the cell width (d) of λ/3. In the designed metasurface, a total of 20 × 20 cells are used to cover the entire surface of the antenna. Figure 19 shows the metasurface configuration, where the phase gradient between adjacent cells along the x-axis is 41°, while along the y-axis, no phase gradient is introduced. It is exhibited that the metasurface is aperiodic in configuration.

The radius of the holes (in mm) of all unit cells of the metasurface for each row from 1 to 20 are as follows: r1 = 0.74; r2 = 0.85; r3 = 0.35; r4 = 0.78; r5 = 0.5; r6 = 0.8; r7 = 0.5; r8 = 1; r9 = 1.32; r10 = 0.66; r11 = 0.84; r12 = 1; r13 = 0.7; r14 = 0.35; r15 = 0.72; r16 = 0.2; r17 = 0.85; r18 = 1.25; r19 = 0.66; r20 = 0.8.

**Figure 20:**
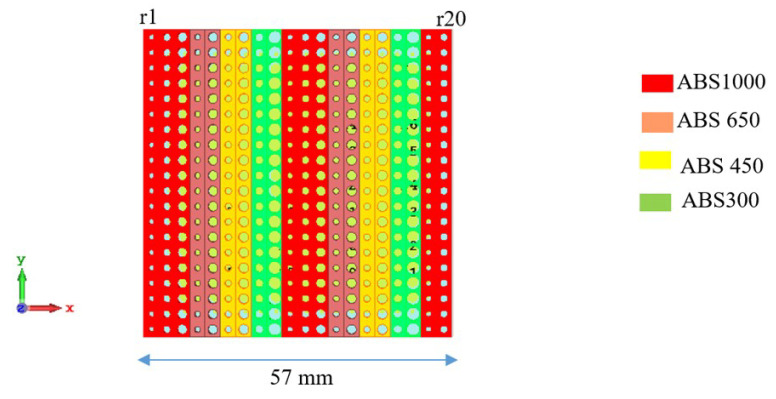
Periodic metasurface configuration.

To make the metasurface structure periodic, the phase gradient between adjacent cells is set to 40°, keeping the overall size the same as the aperiodic metasurface (20 × 20 cells). The steering angle of this metasurface will remain the same as before (i.e., 20°). Figure 20 shows the periodic metasurface configuration. The radius (in mm) of the holes of the periodic metasurface unit cells are: r1 = 0.55; r2 = 0.75; r3 = 0.94; r4 = 0.56; r5 = 0.91; r6 = 0.62; r7 = 0.94; r8 = 0.81; r9 = 1.22; r10 = 0.55; r11 = 0.75; r12 = 0.94; r13 = 0.56; r14 = 0.91; r15 = 0.62; r16 = 0.94; r17 = 0.81; r18 = 1.22; r19 = 0.55; r20 = 0.75. It is noticed that r1–r9 are unique radiuses that repeat periodically.

### 4.3. Periodic and Aperiodic Metasurfaces Performance Comparison

**Figure 21:**
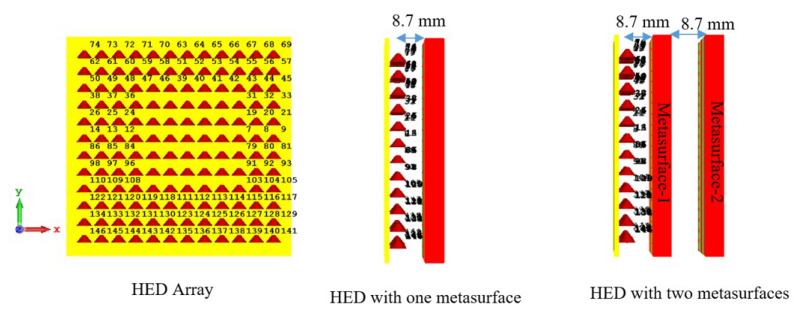
HED array and metasurfaces.

The performance of the periodic and aperiodic metasurfaces are compared to evaluate which metasurface gives better results for the demonstrated antenna. The performance is examined both with one metasurface and two metasurfaces. For performance prediction and parameter tuning, an array of horizontal electric dipoles (HEDs) is used as a feeding base antenna, which needs relatively fewer computational resources whilst having nearly the same radiation characteristics as RCA. Figure 21 shows an HED array with one metasurface and two metasurfaces. For the one-metasurface configuration, the distance from the HED array to the metasurface is kept at 8.7 mm and for the two-metasurface configuration, the distance from the HED array to metasurface-1 is kept at 8.7 mm and the distance between metasurfaces is kept at 8.7 mm. It can be noted that when the two metasurfaces are aligned in the same direction, the maximum beam tilting is realized, while no beam tilting is achieved when they are aligned in the opposite direction.

**Figure 22:**
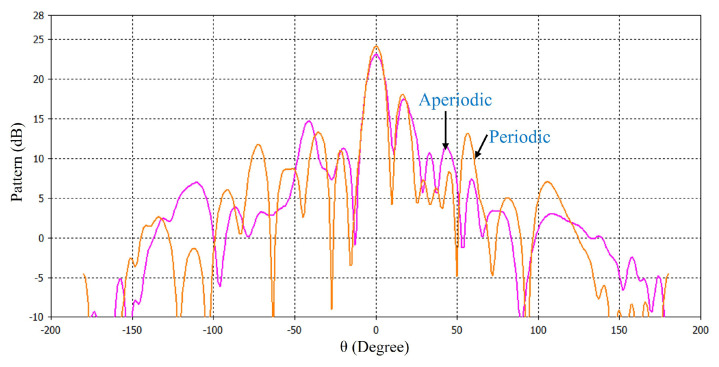
Pattern at 34.5 GHz for HED array and two metasurfaces aligned opposite to each other.

Figure 22 shows the radiation pattern at 34.5 GHz for the HED array and two metasurfaces aligned opposite to each other; the patterns are plotted both for periodic and aperiodic metasurfaces for comparison.

The details of the simulated performance of the periodic and aperiodic metasurfaces are shown in Table 3. In this comparison, the directivity and side-lobe-level (SLL) are taken into consideration. It can be seen that for both one metasurface and two metasurfaces (aligned in the same direction for maximum beam tilting and in the opposite direction for 0° beam tilting cases), the periodic metasurface gives higher directivity and lower SLL. In the demonstrated work design, the periodic metasurface configuration is used due to its better performance over the aperiodic one.

## 5. Metasurface Optimization

To increase the directivity and reduce the side-lobe-level (SLL), the metasurface is optimized by using the optimization algorithms of CST Microwave Studio. The radiuses of the holes of the metasurface cells are optimized by keeping the width and thickness constant. Later, the performance of the RCA array is computed with the optimized metasurface and compared with the unoptimized metasurface performance.

### 5.1. Optimization Procedure

**Figure 23:**
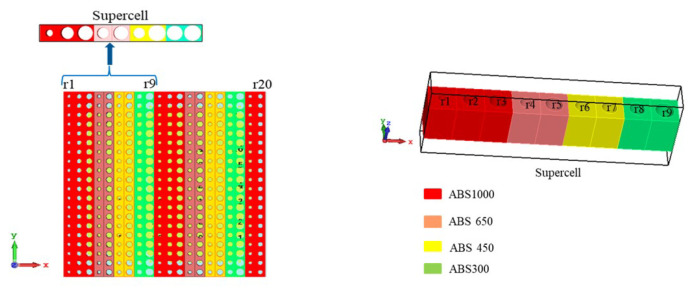
Supercell of the metasurface.

The optimization is conducted with supercell [33] and periodic boundary conditions that mimic an infinitely extended metasurface. A supercell is the combination of unit cells that are repeated periodically to form the metasurface. In the demonstrated metasurface, the combination of the first nine-unit cells is the supercell that is shown in Figure 23. The “CMA Evolution Strategy” algorithm of CST was used for the optimization. The radius of the holes (r1, r2, r3, r4, r5, r6, r7, r8 and r9) of the unit cells were used as the optimization parameters in the algorithm. In the optimizer, the radius limit was set at 0.1–1.4 mm.

**Figure 24:**
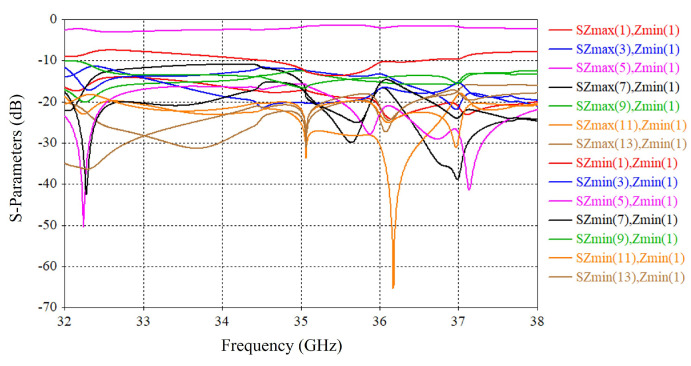
Transmission and reflection modes of the supercell in Floquet analysis.

The step-by-step procedure of the optimization is described as follows:

Step-1: The supercell was simulated in CST MWS under periodic boundary conditions with Floquet port excitation [33]. For this simulation, the supercell was excited with a broadside TE (00) mode propagating along the z-axis. From Floquet analysis, it was exhibited that this supercell supports fourteen propagating TE modes, out of which seven are transmitting and seven are reflecting. Figure 24 exhibits the fourteen propagating TE modes. Among these modes, SZmax(5), Zmin(1) is the main propagating mode, which will be maximized. The other modes are either reflections or deflections in unwanted directions and need to be suppressed.

**Figure 25:**
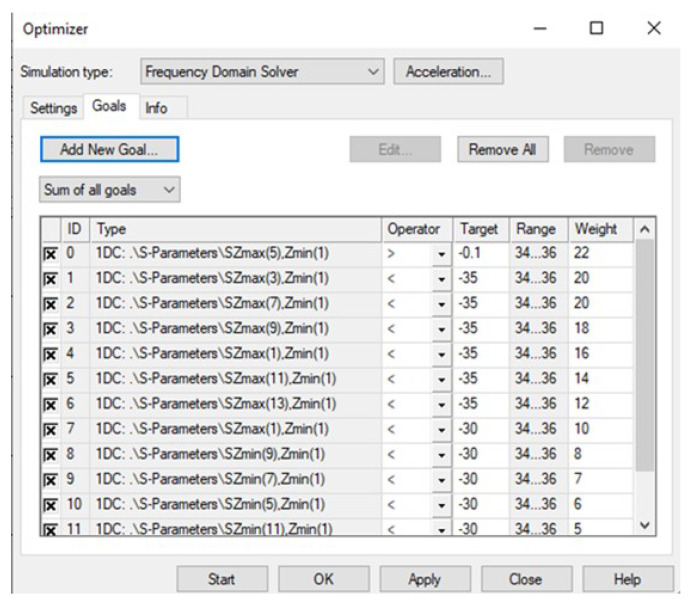
Optimizer settings in CST.

Step-2: In the optimization settings, a target value, frequency range of interest and weight were assigned to each mode. Maximum weight was assigned for the desired mode. For grating modes, the weights were distributed to target the worst grating lobes selectively. Thus, a higher weight value was assigned to the grating modes with high magnitude. The weight values were gradually reduced for a corresponding reduction in the magnitude of the grating modes. Figure 25 depicts the assigned goal for each mode.

**Figure 26:**
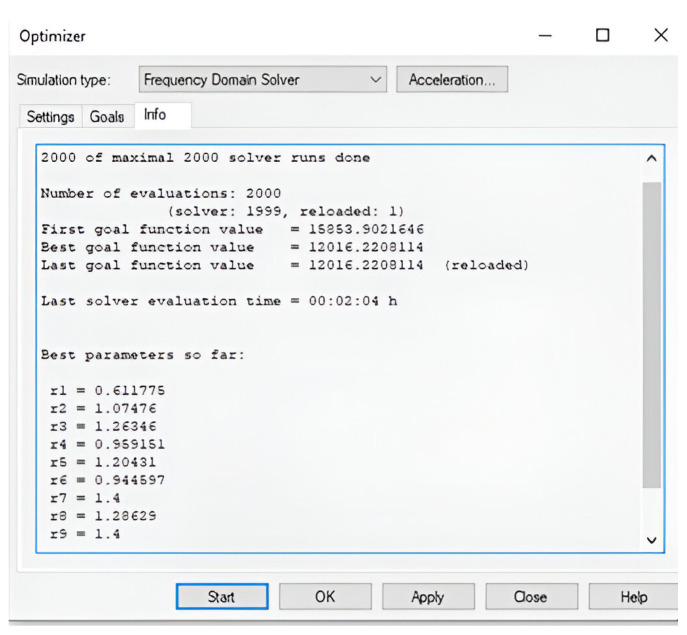
Computed result of the optimizer in CST Microwave studio.

Step-3: The optimizer aims to minimize the goal function value at each iteration. Once the goal function was sufficiently reduced and did not change significantly for at least 10 to 15 runs, then the algorithm was terminated manually. The parameters corresponding to the current best goal function value give the best performance (enhances the desired mode and suppresses the unwanted modes). The best parameter values (radius of the holes) found from the optimizer were used to form the optimized metasurface, which tended to give better performance in terms of directivity and side-lobe-level. The optimizer result is shown in Figure 26. The radius of the holes before and after the optimization are shown in Table 4, which shows a significant change in the hole radius.

### 5.2. Performance Investigation of the Optimized Metasurface

The performance of the optimized metasurface was numerically investigated and compared with the unoptimized metasurface performance. For performance estimation, an HED array was used instead of the actual RCA array to save computation time. The performance was computed when the two metasurfaces were aligned in the same direction (maximum beam titling) and when they were aligned in the opposite direction (0° beam titling). For simulation with the HED array, the distance from the HED array to metasurface-1 was kept at 9.83 mm, and the distance between metasurfaces was kept at 5 mm (from the parametric analysis, it was found that these distances give better performance).

**Figure 27:**
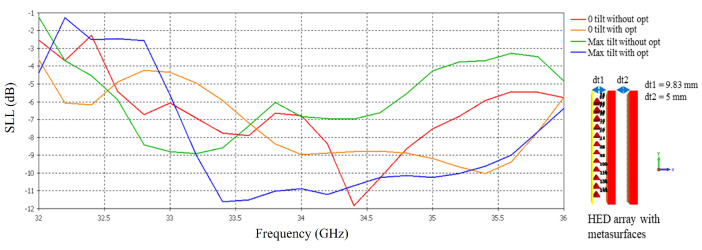
SLL vs. frequency before and after optimization tested with HED array.

Figure 27 shows the SLL vs. frequency of the HED array with two optimized metasurfaces as well as two unoptimized metasurfaces. It can be noticed that when the two metasurfaces are aligned in the opposite direction (0° beam titling), notable improvement in SLL is visible at 33.6–36 GHz for the optimized metasurface, although the minimum value of SLL is better for the unoptimized metasurface, the overall SLL in the entire frequency band is better for the optimized metasurface. For maximum beam titling, a significant improvement in SLL is visible at 33.2–36 GHz for the optimized metasurface.

**Figure 28:**
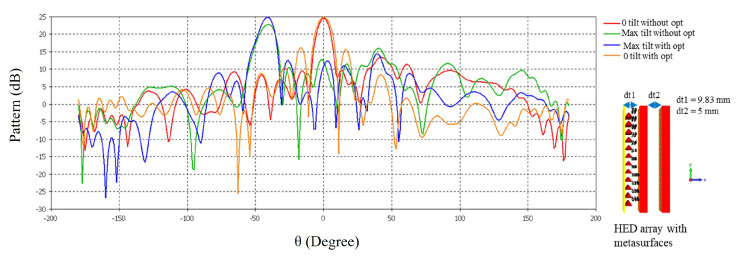
Far-field radiation pattern at 34.5 GHz before and after optimization tested with HED array.

Figure 28 shows the far-field radiation pattern at 34.5 GHz of the HED array with two optimized metasurfaces as well as two unoptimized metasurfaces for maximum and 0° beam tilting. An improvement in maximum directivity is visible with the optimized metasurface; this improvement is more significant for maximum beam tilting, i.e., when the two metasurfaces are aligned in the same direction.

## 6. Testing the Optimized Metasurface Performance with RCA Array

The performance estimation of the optimized metasurface with the HED array has revealed the improvement in directivity and SLL for the beam tilting operation. Now, the optimized metasurface is used with the designed 2 × 2 RCA array, and the beam steering performance is investigated. Performance investigations for maximum beam tilting (when the metasurfaces are aligned in the same direction) and 0° beam tilting (when the metasurfaces are aligned in the opposite direction) are analyzed.

### 6.1. RCA Array with Maximum Beam Tilting Metasurface

**Figure 29:**
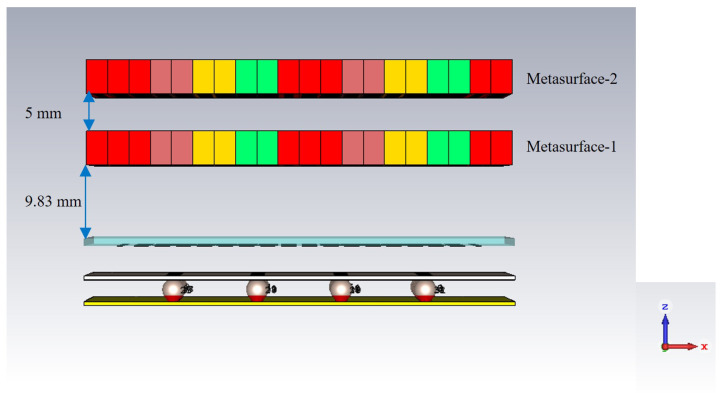
RCA array with two optimized metasurfaces aligned in the same direction.

Figure 29 depicts the designed sphere-fed RCA array and two optimized metasurfaces aligned in the same direction, incorporating maximum beam tilting. The distance from the PRS of the RCA array to metasurface-1 is 9.83 mm, and the distance between the metasurfaces is 5 mm.

**Figure 30:**
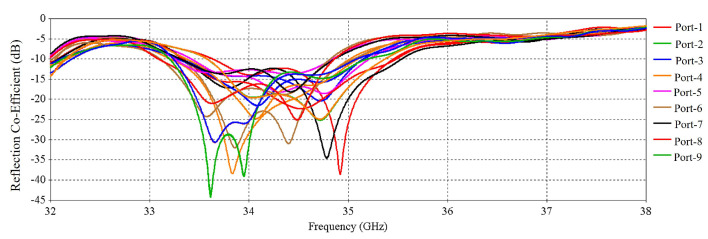
S-parameters of the RCA array with optimized metasurfaces.

Figure 30 shows the active S-parameters of the ports of the RCA array with optimized metasurfaces; there are a total of 32 results for 32 ports. For clarity, nine results are shown here, but some results are exactly the same, so these are skipped. Compared with Figure 11, it is observed that at higher frequencies, the reflection at some ports is increased with the metasurfaces.

**Figure 31:**
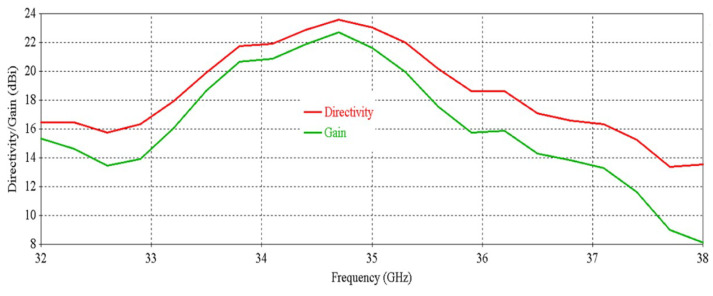
Directivity and gain vs. frequency of the RCA array with optimized metasurfaces.

Figure 31 shows the directivity and gain vs. frequency of the RCA array with two optimized metasurfaces oriented for maximum beam tilting. It is observed that the 3 db directivity bandwidth is 1.94 GHz (33.59–35.53 GHz) and 3 db gain bandwidth is 1.68 GHz (33.66–35.34 GHz).

**Figure 32:**
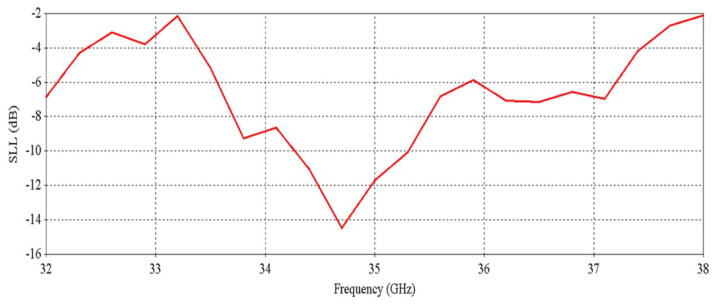
SLL vs. frequency of the RCA array with optimized metasurfaces.

**Figure 33:**
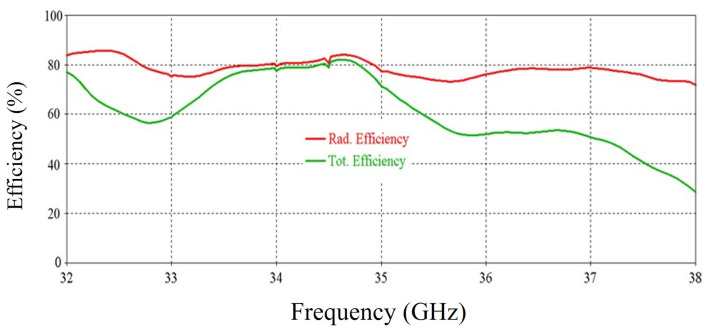
Efficiency vs. frequency of the RCA array with optimized metasurfaces.

Figure 32 and Figure 33 illustrate the results of SLL vs. frequency and efficiency vs. frequency, respectively, for maximum beam tilting. Promising SLL and efficiency can be observed from these results. In Figure 33, a decrease in total efficiency at higher frequencies occurs due to the poor matching at the higher band, as can be seen in Figure 30.

**Figure 34:**
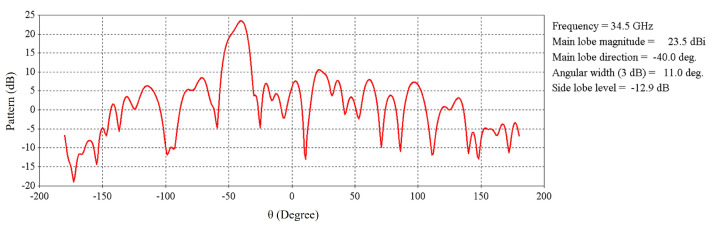
Far-field radiation pattern at 34.5 GHz of the RCA array with optimized metasurfaces for maximum beam tilting.

Figure 34 illustrates a far-field radiation pattern at 34.5 GHz, and the main beam is tilted to 40° as expected.

### 6.2. RCA Array with Two Optimized Metasurfaces Aligned in Opposite Direction (0° Beam Tilting)

**Figure 35:**
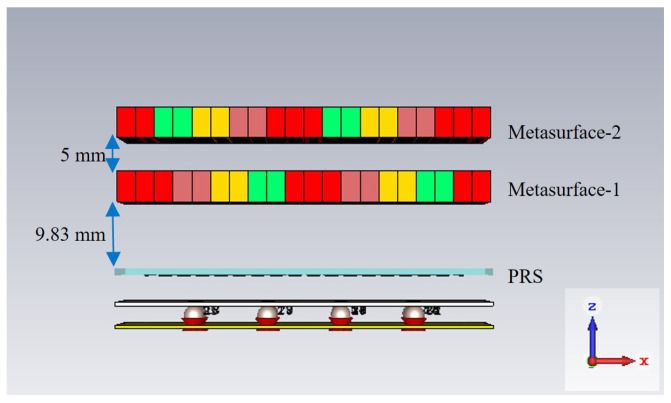
RCA array with two optimized metasurfaces aligned in the opposite direction.

Figure 35 shows the sphere-fed RCA array and two optimized metasurfaces aligned in the opposite direction incorporating 0° beam tilting. The distance from the PRS of the RCA array to metasurface-1 is 9.83 mm, and the distance between metasurfaces is 5 mm.

**Figure 36:**
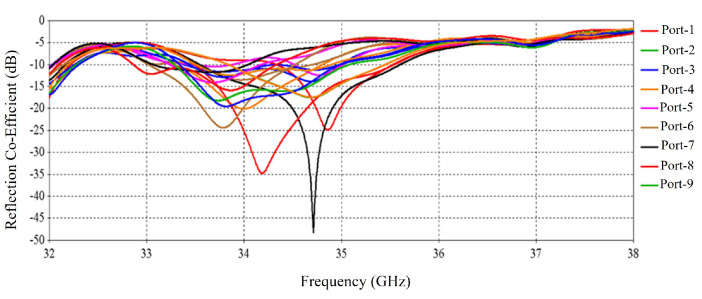
S-parameters of the RCA array with optimized metasurfaces aligned for 0-degree beam tilt.

Figure 36 shows the active S-parameters of the ports of the RCA array. Compared with Figure 11, it is observed that at higher frequency bands, the reflection at some ports is increased after using the metasurfaces. The results are similar to the previous results of Figure 30, where the metasurfaces were aligned for maximum beam tilting.

**Figure 37:**
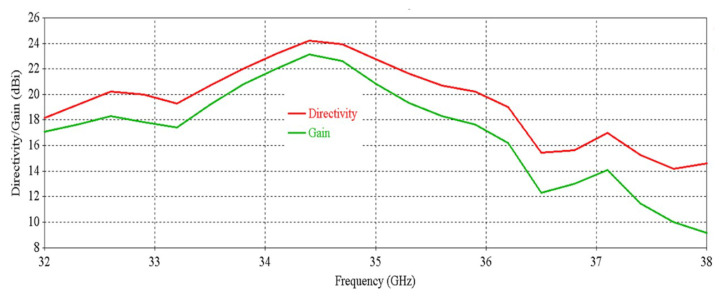
Directivity and gain vs. frequency for 0° beam tilt.

Figure 37 shows the directivity and gain vs. frequency of the RCA array with two optimized metasurfaces positioned for 0° beam tilting. It is observed that the 3 db directivity bandwidth is 1.9 GHz (33.59–35.49 GHz) and the 3 db gain bandwidth is 1.53 GHz (33.66–35.19 GHz).

**Figure 38:**
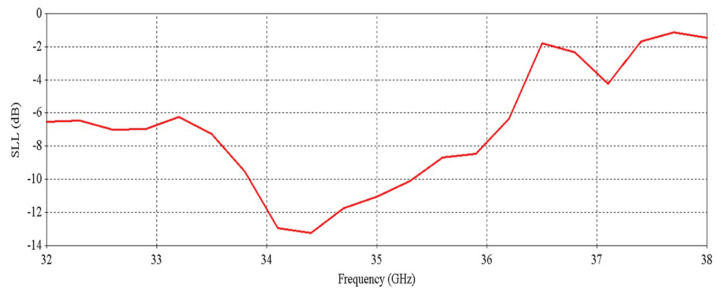
SLL vs. frequency for 0° beam tilt.

**Figure 39:**
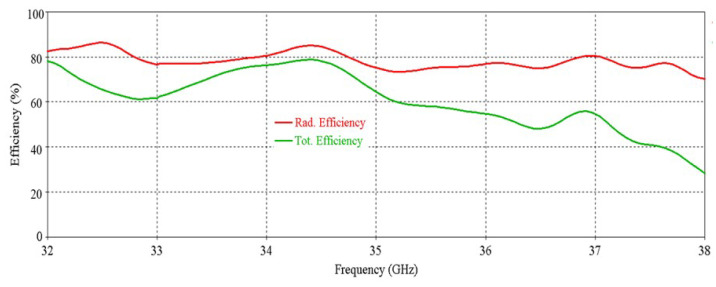
Efficiency vs. frequency for 0° beam tilt.

Figure 38 and Figure 39 illustrate the results of SLL vs. frequency and efficiency vs. frequency, respectively. Satisfactory SLL and efficiency can be observed from these results. In Figure 39, a decrease in total efficiency at higher frequencies happens due to the poor matching at higher bands, as can be seen in Figure 36.

**Figure 40:**
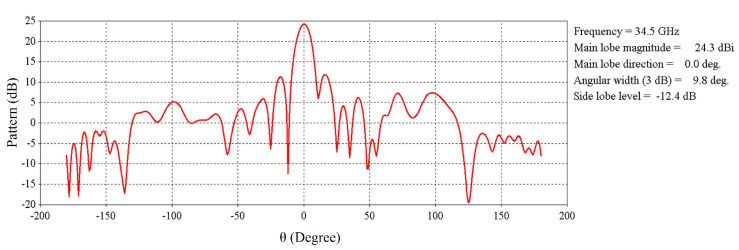
Far-field radiation pattern at 34.5 GHz for 0° beam tilt.

Figure 40 illustrates the far-field radiation pattern at 34.5 GHz, and the main beam is directed toward the broadside as expected.

### 6.3. Performance Comparison Between Maximum and 0° Tilt for Different Metasurface Orientations

The performances of the RCA array for two orientations of the metasurfaces (aligned in the same direction for maximum beam tilting and aligned in the opposite direction for 0° beam tilting) are compared to demonstrate the performance in different beam tilting positions.

**Figure 41:**
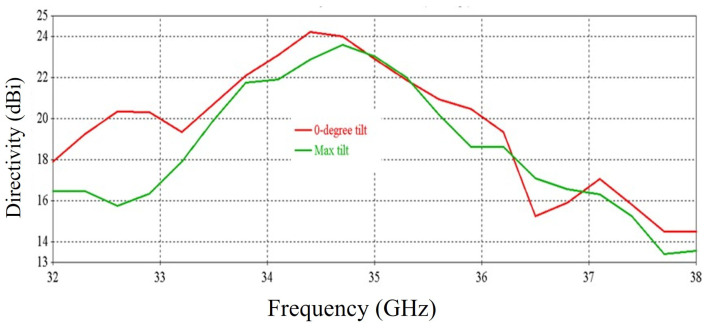
Directivity comparison for 0° and maximum beam tilt.

**Figure 42:**
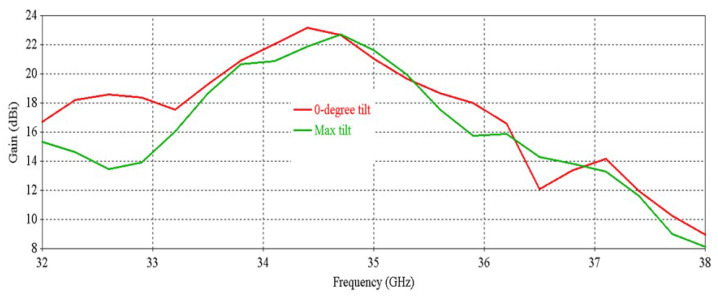
Gain comparison for 0° and maximum beam tilt.

**Figure 43:**
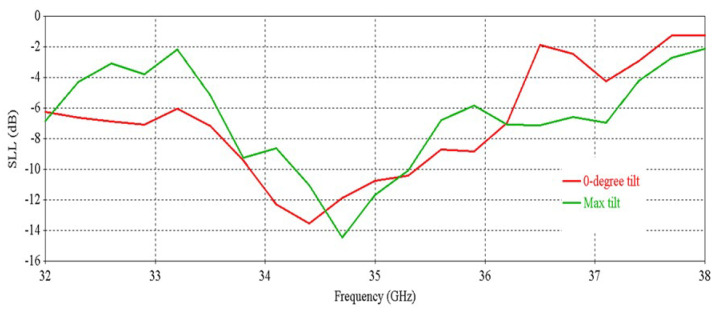
SLL comparison for 0° and maximum beam tilt.

Figure 41, Figure 42 and Figure 43 exhibit the performance comparison of directivity, gain and SLL, respectively, for 0° and maximum beam tilting cases. The 3 db directivity bandwidth is 1.94 GHz (33.59–35.53 GHz) for maximum beam tilting, whereas it is 1.9 GHz (33.59–35.49 GHz) for 0° beam tilting. It is exhibited that 3 db directivity bandwidth is nearly the same for maximum and 0° beam tilting. The 3 db gain bandwidth is 1.68 GHz (33.66–35.34 GHz) for maximum beam tilting, whereas the 3 db gain bandwidth is 1.53 GHz (33.66–35.19 GHz) for 0° beam tilting. It is exhibited that the 3 db gain bandwidth is slightly higher for maximum beam tilting than 0° beam tilting. The SLL comparison of Figure 43 indicates that the 0° beam tilting orientation metasurface has a better performance than the maximum beam tilting metasurface at lower frequencies, but at higher frequencies, maximum beam tilting has a better SLL performance.

Table 5 depicts the performance comparison of the proposed antenna with the state-of-the-art works. It can be noticed that the proposed antenna has the highest gain, and it does not require any DC power supply due to the utilization of the mechanical beam steering technique. Moreover, the antenna has a very good scanning angle. The most prominent features of our demonstrated system are its in-antenna power combining capability for chip-fed high-power applications in millimeter-wave band, utilization of passive metasurface for the beam steering, ability to 3D print the metasurfaces, low thickness, excellent gain and scanning range. The optimized metasurface and the designed antenna provide an excellent system of microwave power combining and wide-angle beam steering.

## 7. Antenna Performance with Closely Spaced Metasurfaces

**Figure 44:**
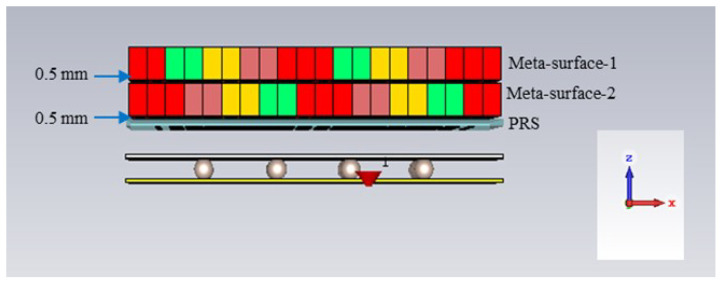
RCA array with lossy feed distribution network and closely spaced metasurfaces for 0° beam tilting.

The performance of the RCA array (with a lossy feed distribution network) is assessed when the metasurfaces are placed very close to the antenna and closely placed near each other. In this investigation, the distance from the PRS of the RCA array to first metasurface was kept at 0.5 mm and the gap between metasurfaces was kept at 0.5 mm. The metasurface orientation for the 0° beam tilting scenario was observed. Figure 44 shows the antenna configuration with closely spaced metasurfaces.

**Figure 45:**
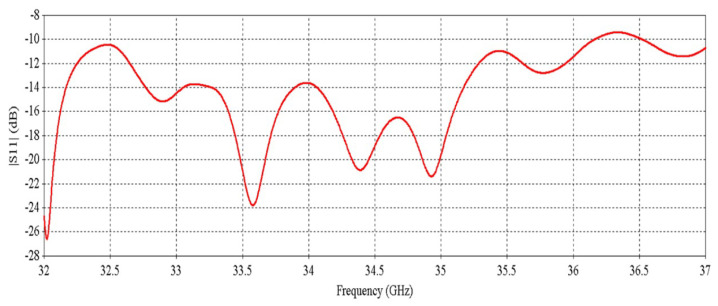
Input reflection coefficient of the RCA array with closely spaced metasurfaces.

Figure 45 shows the input reflection coefficient at the input port of the feed distribution network, which demonstrates very low reflection in the entire observed frequency band from 32 to 37 GHz.

**Figure 46:**
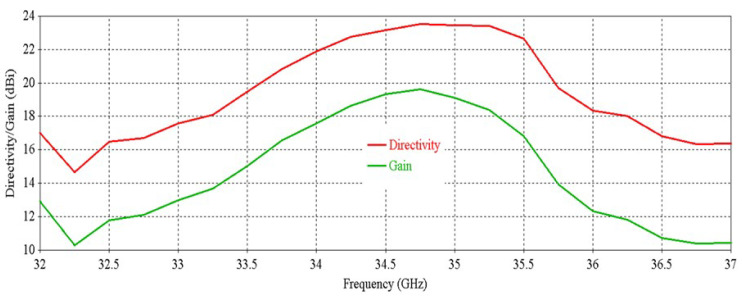
Directivity and gain vs. frequency of the RCA array with closely spaced metasurfaces.

The directivity and gain of the RCA array with closely spaced metasurfaces and fed through a feed distribution network are illustrated in Figure 46. The 3 db directivity bandwidth is 2.03 GHz (33.67–35.7 GHz), and the 3 db gain bandwidth is 1.76 GHz (33.76–35.52 GHz). These results are similar to the results when the metasurfaces were placed further apart than this closely spaced configuration (i.e., 9.83 mm from RCA array to metasurface-1 and 5 mm distance between metasurface-1 and metasurface-2).

**Figure 47:**
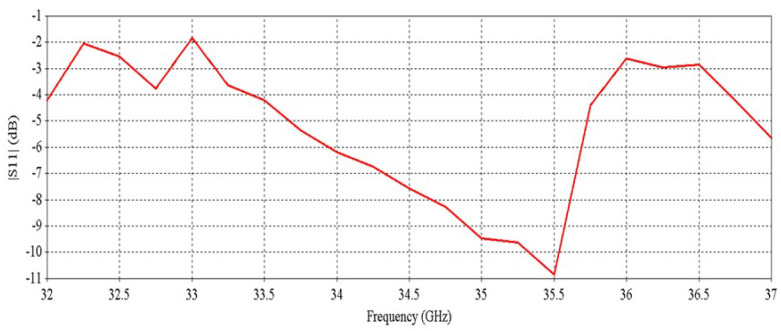
SLL vs. frequency of the RCA array with closely spaced metasurfaces.

The SLL result shown in Figure 47 demonstrates that the side-lobe-level performance is poor for the closely spaced metasurfaces configuration compared to the previous results.

**Figure 48:**
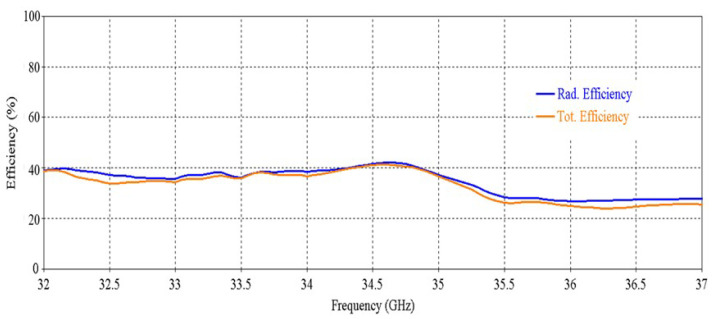
Efficiency vs. frequency of the RCA array with closely spaced metasurfaces.

Figure 48 demonstrates the efficiency of the RCA array for closely spaced metasurfaces, which shows poor performance compared to the previous results.

**Figure 49:**
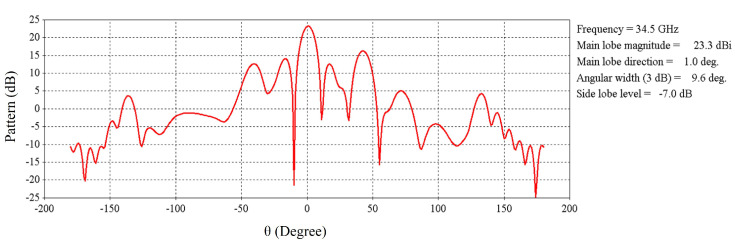
Pattern at 34.5 GHz of the RCA array for 0° beam tilting with closely spaced metasurfaces.

**Figure 50:**
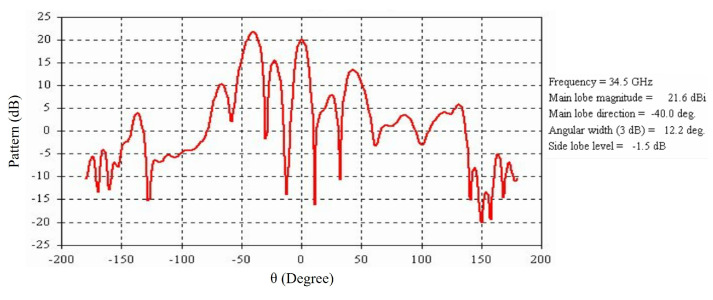
Pattern at 34.5 GHz of the RCA array for maximum beam tilting with closely spaced metasurfaces.

Figure 49 and Figure 50 plot the far-field radiation pattern of the RCA array with closely spaced metasurfaces at 34.5 GHz for 0°, and for maximum beam tilting, respectively, the results show the presence of high grating lobes.

Therefore, it is explicit that a closely spaced metasurface configuration can be used with the appearance of a high grating lobe and reduced efficiency. However, this configuration is useful where compact beam scanning system is required.

## 8. Conclusions

The demonstrated RCA array can be a promising candidate for a chip-fed in-antenna power combining technique for millimeter-wave high gain and low-loss applications. The investigated planar dielectric phase gradient metasurface can steer the beam over a wide scanning range. The explored technique is an effective solution for a lightweight, low-profile and low-cost design of a passive beam steering system, which is free from thermal loss associated with active electronic components, non-linear distortion, and sensitivity to temperature variation. The explored optimization procedure of the metasurface is an important investigation for designing an accurate wide-angle and wideband beam steering system with the best achievable performance. A detailed analysis of the chip-fed RCA array with a metasurface could be a promising source of information for designing a passive beam steering system with wide scanning range for a millimeter-wave high-gain high-power antenna system for electronic sensing and spectral monitoring applications. 

## Figures and Tables

**Table 1:** Dimensions of the antenna element.

Parameter	Value (mm)	Parameter	Value (mm)	Parameter	Value (mm)
A	11.314	B	1.575	C	1.138
E	0.375	F	1.3735	G	1.592
H	0.31	I	0.85	J	1.338
K	0.125	L	3	M	2.82
N	3.92	O	35	P	1.8546
Q	0.4	R	1.48	D1	2.667
D2	2.663	D3	2.62	D4	2.04
D5	0.68	T1	2.835	T2	8.506
T3	14.176	T4	19.847	T5	25.517

**Table 2:** Properties of the dielectric materials.

Material	Dielectric Constant	Loss Tangent	Component	Thickness/Diameter (mm)
Alumina	9.98	0.0002	Sphere	3
Ultralum 3850	2.9	0.0025	Circuit board	0.05
Rogers RT5880	2.2	0.0009	Middleboard	0.508
TMM4	4.7	0.002	Superstrate	1.03

**Table 3:** Period and aperiodic metasurface performance comparison.

Configuration	SLL (dB)	Directivity (dB)
HED array only	−13.2	26.7
One periodic metasurface	−9.5	25.9
One aperiodic metasurface	−7.4	24.9
Two periodic metasurfaces aligned in the same direction	−4	21.9
Two aperiodic metasurfaces aligned in the same direction	−3.4	21.7
Two periodic metasurfaces aligned in the opposite direction	−6.1	24.1
Two aperiodic metasurfaces aligned in the opposite direction	−5.5	23.1

**Table 4:** Radiuses of the holes before and after optimization.

Cell	Hole Radius (mm) Before Optimization	Hole Radius (mm) After Optimization	% Change
1	0.55	0.61	10.91
2	0.75	1.07	42.67
3	0.94	1.26	34.04
4	0.56	0.96	71.42
5	0.91	1.2	31.87
6	0.62	0.94	51.61
7	0.94	1.4	48.94
8	0.81	1.3	60.49
9	1.22	1.4	14.75

**Table 5:** Performance comparison of the proposed antenna with the state-of-the-art works.

Ref	Freq (GHz)	Steering Technique	Scanning Angle (°)	Peak Gain (dBi)	DC Power
[8]	11	Mechanical	±46	19.4	No
[16]	5.5	PIN Diode	±36	7	Low
[19]	9.375	Mechanical	±20	-	No
[32]	35	Mechanical	±40	21.5	No
[34]	2.62	Water	±20	5.7	No
[35]	30	Mechanical	±39	16	No
[36]	11	Mechanical	±57	19.9	No
This work	35	Mechanical	±40	25.03	No

## Data Availability

The original contributions presented in this study are included in the article. Further inquiries can be directed to the corresponding author(s).
